# Relevant determinants of Dutch patients’ informed decision-making and use of online access to medical records

**DOI:** 10.1093/heapro/daae071

**Published:** 2025-02-21

**Authors:** Rosa R L C Thielmann, Ciska Hoving, Jochen W L Cals, Rik Crutzen

**Affiliations:** Faculty of Health, Medicine and Life Sciences, Department of Health Promotion, School CAPHRI, Care & Public Health Research Institute, Maastricht University, P. Debyeplein 1, HA 6226, Maastricht, The Netherlands; Faculty of Health, Medicine and Life Sciences, Department of Health Promotion, School CAPHRI, Care & Public Health Research Institute, Maastricht University, P. Debyeplein 1, HA 6226, Maastricht, The Netherlands; Faculty of Health, Medicine and Life Sciences, Department of Family Medicine, School CAPHRI, Care & Public Health Research Institute, Maastricht University, P. Debyplein 1, HA 6226, Maastricht, The Netherlands; Faculty of Health, Medicine and Life Sciences, Department of Health Promotion, School CAPHRI, Care & Public Health Research Institute, Maastricht University, P. Debyeplein 1, HA 6226, Maastricht, The Netherlands

**Keywords:** informed decision-making, patient empowerment, patient participation, electronic health records, health records, personal, medical records, patient access to records

## Abstract

Patient online access to medical records can foster patient empowerment and advance patient-centered healthcare. Despite high patient interest, user rates often remain low. While previous research has identified determinants influencing the adoption of online access, this study assessed the relevance of these determinants. By doing so, this study aimed to point towards measures to improve adoption efficiently. Furthermore, supporting patient-informed decision-making about using online access might facilitate sustained use. Therefore, conducting a nationwide online survey study among Dutch adults, we examined the relevance of 22 psychological determinants for the use of online access (*N* = 1888) and decisional conflict (as an indicator of uninformed decision-making) regarding use (*N* = 3403). Analyses concerned visualization of univariate distributions of determinants and their associations with (i) use and (ii) decisional conflict regarding use. Greater relevance was indicated by lower means and stronger associations. Results showed that secured data privacy and feeling sufficiently instructed were most relevant for use. Concerning decisional conflict regarding the use, additionally, patients’ beliefs about the effects of online access on their ability to participate in their healthcare process and on the patient–provider relationship were most relevant. Overall explained variance was low (*R*^2^ = 0.17 for use and *R*^2^ = 0.19 for decisional conflict). Efficiently supporting the use of online access and informed decision-making about use might be achieved by addressing data privacy, providing clear instructions and communicating potential effects. The low explained variance indicates the need for additional measures, such as facilitating easy opportunities and understanding individual patient preferences.

**Contribution to Health Promotion**

- By identifying the most relevant determinants, the study suggests efficient measures to enhance the adoption of online access.
- Supporting patients in making informed decisions about using online access might enhance the likelihood of sustained use.
- Improved adoption and sustained use of online access increases the likelihood of potential benefits, such as patient empowerment and participation.

## INTRODUCTION

Patient participation gradually became a focus in efforts to improve health service delivery and care quality worldwide (Castro *et al*., 2016). Patient online access to their medical records (hereafter ‘online access’) may empower patients to be more involved in their own care (Irizarry *et al*., 2015; Benjamins *et al*., 2021). In July 2020, patients in the Netherlands became legally entitled to receive their general practice medical record electronically on request (Ministry of Health, Welfare and Sport, 2017), which is mostly facilitated via online patient portals. In those portals, patients can view medication and allergy lists, medical notes and diagnostic test results (HealthIT, 2019). Although 88% of Dutch patients considered it important to have online access to their medical data at the time of introduction (Netherlands Patients Federation, 2020), only 14% had ever accessed their medical record 2 years later (OPEN-Eerstelijn, 2022). This discrepancy of high patient interest yet low user rates, usually between 15% and 30%, was also observed in various other countries (Dhanireddy *et al*., 2014; Lyles *et al*., 2020).

Challenges in the adoption and use of online access are common factors related to medical practice and environment, technology, or patient characteristics (Niazkhani *et al*., 2020). In previous research about Dutch patients’ needs and expectations regarding online access, participants identified challenges pertaining to a lack of instruction, technological difficulties with the portal and concerns about data privacy and complex medical language in their record. However, participants were eager to use online access as they expected benefits from use, such as a better overview about their healthcare and appointments, feelings of empowerment and improved communication with their general practitioner (GP) (Thielmann *et al*., 2023). Similarly, expected benefits are frequently highlighted as crucial reasons for patients to (start) using online access (McGinn *et al*., 2011; Crameri *et al*., 2020). However, patients also expect disadvantages such as feelings of distress and anxiety when receiving sensitive or incomprehensible medical information through technology without the presence of a healthcare provider (Jilka *et al*., 2015; Baun *et al*., 2020). Furthermore, previous findings indicate that older adults (Logue and Effken, 2012; Lyles *et al*., 2020), patients with limited health and digital literacy (Emani *et al*., 2012; Lyles *et al*., 2020) and patients with a lower socioeconomic status (Roblin *et al*., 2009; Emani *et al*., 2012) were more prone to face difficulties in accessing and meaningfully engaging with their medical data. In addition, while patients with a lower socioeconomic status or who are members of ethnic minorities might be less likely to use online access (Roblin *et al*., 2009; Yamin *et al*., 2011), these same groups often report experiencing more benefits from online access. For example, they more often report that by using online access they feel better informed about their care (Gerard *et al*., 2017) and feel more positive about their doctor (Bell *et al.*, 2017).

Evidently, many factors play a role in patients’ use of online access (Prey *et al*., 2016). Those factors are commonly labeled as ‘determinants’ and, in the context of this study, we define them as psychological constructs that are assumed to causally contribute to patients accessing their records online. Strategies that accompany the introduction of online access to support the initial use among patients would ideally address all those determinants. However, in practice, there are constraints regarding content development and delivery (e.g. time, budget, staff availability). Therefore, it is important to identify and address those determinants that are most relevant to address (Crutzen *et al*., 2017), i.e. determinants that show the strongest association with the use of online access *and* the greatest room for improvement. Addressing these determinants is likely to yield the most substantial effects. Additionally, patients should be enabled to make an informed decision about whether they want to use online access. Informed decision-making is the process resulting in decisions that the patient makes based on relevant and good quality knowledge, that reflects the patient’s values and that results in implementing their decision (i.e. accessing their online records or not) (Bekker *et al*., 1999; Marteau *et al*., 2001). When making an informed decision, people are less likely to experience uncertainty or regret about their decision, which is called ‘decisional conflict’ (O’Connor, 1995; Knops *et al*., 2013), and they are more likely to continue the chosen option (Sun, 2005). Strategies aimed at decreasing decisional conflict patients experience when deciding whether to use online access might therefore lead to more sustained use and thereby facilitate potential benefits, such as increased patient engagement in healthcare.

The relevance of determinants for patients’ online access use and experienced decisional conflict about the decision whether to use online access are yet unknown. Consequently, this study aims to identify the most relevant determinants for Dutch patients by examining both their room for improvement and their association with (i) the use of online access and (ii) decisional conflict about the decision whether to use online access.

## METHODS

### Research design

This research was the first part of a larger project, which was preregistered in the Open Science Framework (OSF): 3gnx2. For this study, cross-sectional data were collected in July 2021 via an online survey. Data collected in this study were pseudonymized before analyses, meaning that the research team could not identify specific persons within the dataset (Crutzen *et al*., 2019). The study was approved by the Maastricht University Faculty Research Ethics Committee (approval number: FHML-REC/2021/071). The STROBE guidelines for observational research were followed for reporting (Von Elm *et al*., 2007). Non-identifiable data can be requested and the survey, analysis scripts and output of the analysis (in high resolution) are available at OSF.

### Participants and recruitment

Participants were recruited from panel members of Flycatcher Internet Research (2020), a Dutch ISO-certified research agency. The sample size was calculated based on the desired number of participants remaining at the end of the larger project, the anticipated dropout rate throughout the project period and the expected effect size for the analyses planned for the final stage of the larger project. The analyses planned for the final stage were on the effect of the user group (patients who have never used online access, patients who have used it before the study and patients who recently started using it) on within-person changes in effect measures over a 1-year period. As we could not infer the effect size from earlier research, we assumed a small effect size of Cohen’s *d* = 0.2 and aimed for a margin of error (half-width) of 0.1 and a confidence level of 95%. These parameters were included in the sample size calculation using the userfriendlyscience (*ufs)* package (Peters and Crutzen, 2021) in R (R Core Team, 2013). We aimed for a total sample size of 3460 participants for this study.

As a preparatory step, panel members who met the inclusion criteria of having been in contact with the general practice in the past 6 months were identified, as we were interested in recent experiences. Subsequently, a sample representative for this group based on age, gender, education and location was invited to participate in this study. Within a week, the sample received one invitation and two reminders per email. Informed consent was obtained online. Completing the survey took 15 min on average. Participants were reimbursed in the form of panel points, worth about 2 euros (2.14 US dollars), which they could exchange for gift vouchers.

### Measurements

Survey items relevant to this study concerned (i) socio-demographic characteristics; (ii) outcome measures, i.e. use of online access and decisional conflict regarding use and (iii) determinants, i.e. beliefs about online access. The pretesting of the survey took place with both native and second-language Dutch speakers.

#### Socio-demographic characteristics

Socio-demographic characteristics that earlier research showed to potentially impact the use of online access were assessed: Age, gender identity, educational level, migration background, province, digital and health literacy, presence of a chronic illness, health status and GP visit due to a psychological complaint. The highest completed educational level was categorized as low (e.g. primary education), intermediate (e.g. secondary vocational education) and high (e.g. university education) (Statistics Netherlands (CBS), 2022a). A participant was considered to have a migration background if they were born abroad (Statistics Netherlands (CBS), 2022b). Digital literacy was assessed with five items from a Dutch measurement tool developed to identify a patient’s digital literacy in general practice (Pharos (Dutch Centre of Expertise on Health Disparities), 2021). Items asked, e.g. ‘Do you sometimes use an app?’ and answer options and scores were ‘no’ (0), ‘with help of for example family or friends’ (2) and ‘yes’ (4). All item scores were summed, divided by 5 and multiplied by 25. Digital literacy sum scores ranged from 0 [low digital literacy] to 100 [high digital literacy]. To get a multifaceted yet concise indication of health literacy, six items covering all cognitive domains deemed necessary to handle health information within the healthcare setting (Finbråten *et al*., 2018) were chosen from the HSL-EU-Q47 (Rademakers *et al*., 2020). Items were formulated as questions (e.g. ‘How easy would you say it is to find information on treatment of illnesses that concern you?’). Responses and scores were ‘very easy’ and ‘easy’ (1) and ‘difficult’ or ‘very difficult’ (0) (Rademakers and Heijmans, 2018). Sum scores ranged from 0 [low health literacy] to 6 [high health literacy].

#### Outcomes: use of online access and decisional conflict regarding use

Given that the statutory right for patient access in the Netherlands was introduced just 1 year before the survey, use in this study is defined as having accessed the general practice medical record online at least once. After participants received written and video explanation about online access, they were asked whether they had ever used online access (Yes/No). The Decisional Conflict Scale (O’Connor, 1993) was used to measure decisional conflict. The low literacy version was used as it is recommended for those with limited reading or response skills and is therefore more suitable to apply in inclusive research. The validated English language scale (Linder *et al*., 2011; Garvelink *et al*., 2019) was translated into Dutch and adapted to the research topic by the research team. Ten items inquired about whether the participant (i) felt informed about the two options (i.e. using online access or not) and their benefits and disadvantages; (ii) had clarity about own personal values regarding the two options and (iii) felt supported in making a choice or pressured to choose a course of action. Answer categories and scores were ‘yes’ (0), ‘unsure’ (2) and ‘no’ (4). All item scores were summed, divided by 10, multiplied by 25 and divided by 20 (O’Connor, 1993). Sum scores ranged from 0 [no decisional conflict] to 20 [extremely high decisional conflict].

#### Determinants: beliefs about online access

Twenty-two items assessed psychological determinants, i.e. beliefs, related to online access. The content of the items was derived from a preceding interview study in which participants had identified needs and expectations regarding the use of online access (Thielmann *et al*., 2023). Those needs and expectations were, when applicable, matched with pre-defined psychological constructs. The constructs, supplemented with constructs identified in previous research on online access to medical records (listed in the introduction), were operationalized by following instructions from the PsyCoRe (2020). We acknowledge the usefulness of specific theories to operationalize psychological constructs. However, we derived psychological constructs from multiple theories, as we believe this is needed in a problem-driven context. Perceived ability to use online access, perceived approval of online access used by the GP, perceived importance of personal contact and perceived sufficiency of instruction were each assessed by one separate item. Additionally, two items assessed expectations regarding secured data privacy, five items measured expected changes in the GP–patient relationship and emotions, eight items focused on expected practical changes in healthcare and three items related to expected influence on decision-making within healthcare. All beliefs were assessed on bidimensional 7-point Likert scales embedded in the statements with the left anchor being the lesser/lower/worse assessment and the right more/higher/better assessment of a belief (e.g. ‘I find viewing my health information online (1) unsafe—(7) safe’).

### Data analysis

Descriptive analyses were conducted using IBM SPSS Statistics 28. Chi-square tests were performed to determine whether the proportion of participant characteristics measured with categorical variables was equal and *t*-tests were performed to determine whether means of characteristics measured with continuous variables were equal between participants that had ever or had never used online access. The main analysis was done in R (R Core Team, 2013). The visualization method Confidence Interval-Based Estimation of Relevance (CIBER) (Crutzen *et al*., 2017) was used to establish the relevance of determinants for the outcomes use of online access and decisional conflict regarding use, respectively. The CIBER method visualizes (i) univariate distributions of each determinant and (ii) associations with outcomes (based on correlations). The function *binaryCIBER* for dichotomous outcome measures was used for the use of online access, and the function *CIBER* for continuous outcome measures was used for decisional conflict. Both functions are part of the *behaviorchange* R package. For both univariate distributions and correlations, confidence intervals (represented by the horizontal ends of diamond shapes) provide a measure of the precision with which these can be estimated [in contrast to *p*-values (Peters and Crutzen, 2018)]. The CIBER diamond plots depicting these data allow for comparison between determinants in terms of relevance: univariate distributions show room for improvement (defined as the difference between the sample mean and the favorable end of the distribution) for each determinant. Simultaneous visualization of the association strength of each determinant with the outcome facilitates comparison of the relevance determinants have for that outcome. Greater relevance is established through the comparison with other determinants, considering both the room for improvement and the strength of associations.

By nature, a relationship between patients’ use of online access and beliefs about it can only exist for participants who were aware of the possibility of online access prior to the study. Therefore, the relevance of beliefs for the use of online access was analyzed in a subsample consisting of participants that stated they knew about the possibility of online access prior to the study.

## RESULTS

### Participant characteristics

In this study, 3403 people participated of whom 1133 (33%) had used online access at least once. Table 1 describes the characteristics of participants.

**Table 1:** Participants’ characteristics

Characteristic	Total		Users		Non-users		*p* value
	*n*	%	*n*	%	*n*	%	
Aware of online access prior to study	1,888	55.5	1,133	100.0	755	33.3	** < 0.001**
Gender							0.69
Women	1,675	49.2	555	49.0	1120	49.3	
Men	1,718	50.5	576	50.8	1142	50.3	
Another gender/nonbinary	10	0.3	2	0.2	8	0.3	
Education level							0.12
Low	930	27.3	281	24.8	649	28.6	
Medium	1,598	47.0	561	49.5	1037	45.7	
High	875	25.7	291	25.7	584	25.7	
Migration background	147	4.3	104	4.6	43	3.8	0.09
Chronic disease presence	1,279	37.6	491	43.3	788	34.7	** < 0.001**
Ever visited GP due to psychological complaint	1,301	38.2	494	43.6	807	35.6	**0.02**
	Mean	SD	Mean	SD	Mean	SD	
Decisional conflict [range 0–20]	4.33	4.15	3.24	3.49	4.87	4.34	**0.02**
Age [years]	49.91	17.13	49.61	17.12	50.06	17.14	0.56
Health literacy (HL) [range 0–6]	5.42	1.22	5.38	1.33	5.44	1.16	0.43
Digital literacy (DL) [range 0–100]	92.36	17.10	93.79	15.76	91.65	17.69	0.57

Note. *N* = 3403. Testing equality of means was done with *t*-tests. Testing equal frequency distribution was done with Pearson chi-square tests. *p* ≤ 0.05 is significant and presented in bold.

### Relevance of determinants

**Fig. 1:**
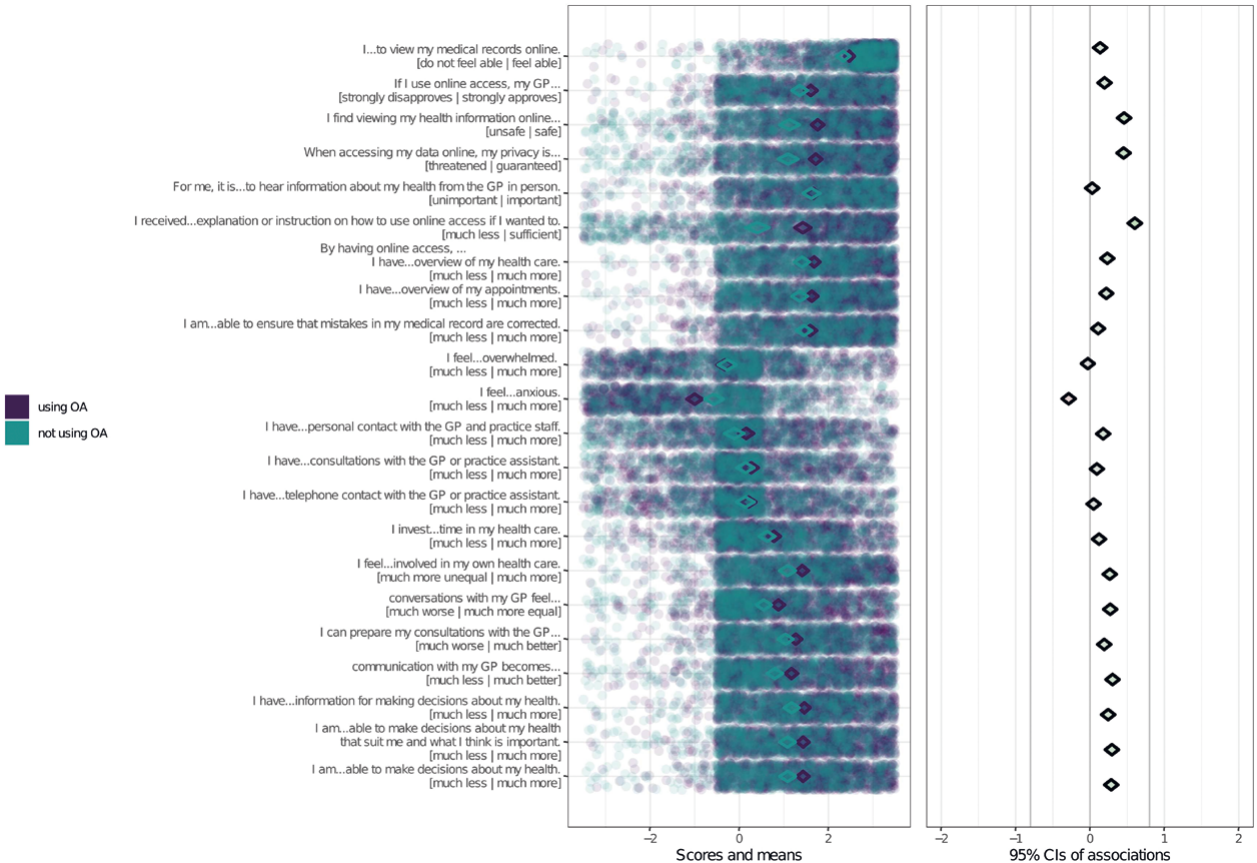
Confidence interval-based estimation of relevance (CIBER) plots for univariate distributions of scores on beliefs for users and non-users [left panel] and associations of determinants with use of online access [right panel]. Nagelkerke *R*^2^ = 0.17. *N* = 1888.

**Fig. 2:**
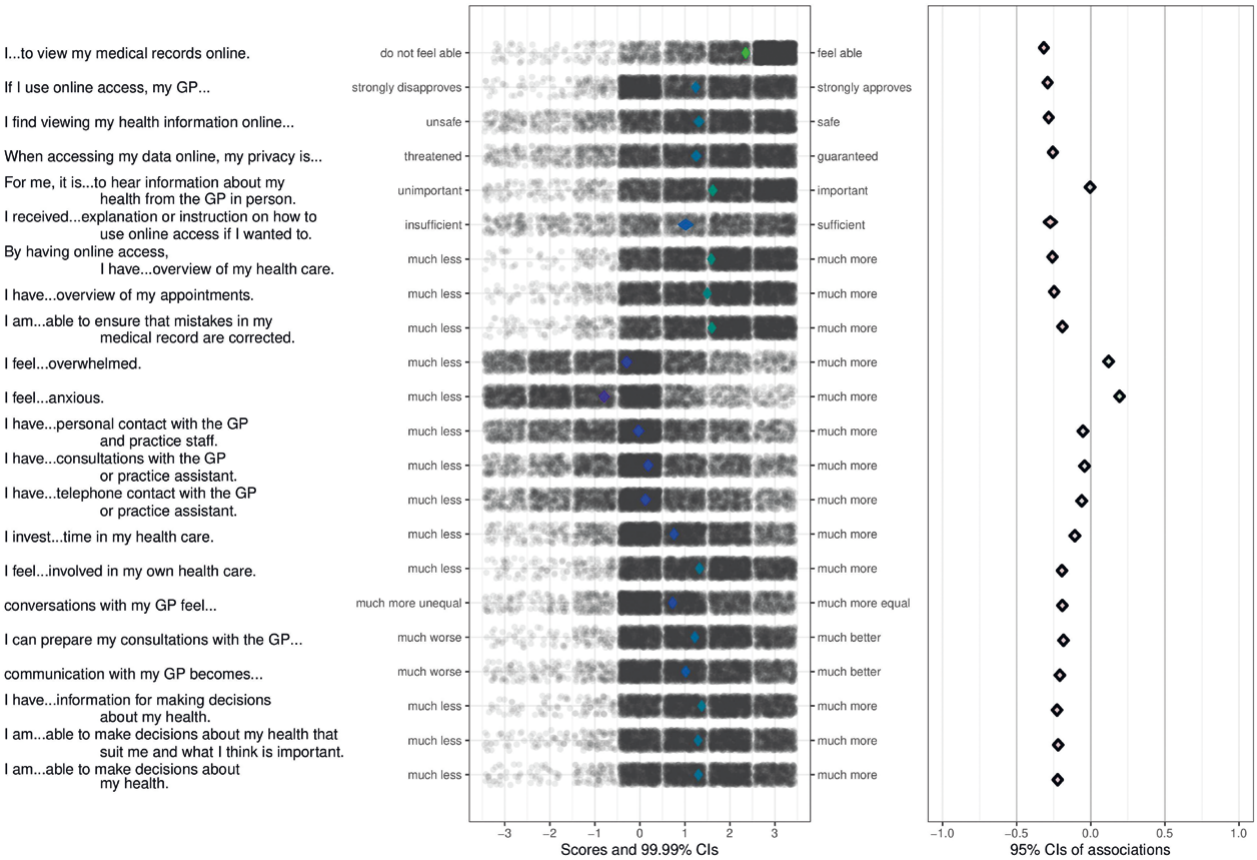
Confidence interval-based estimation of relevance (CIBER) plots for univariate distributions of scores on beliefs [left panel] and associations of determinants with decisional conflict [right panel]. Adjusted *R*^2^ = 0.19. *N* = 3403.

Figures 1 and 2 show the results of the CIBER approach for, respectively, the use of online access and decisional conflict. The items that were used to assess the beliefs plus anchors of the items are presented next to the left panel. In the left panel, item scores of individual participants are shown as dots with jitter added to prevent overplotting. Diamonds in the left panel show the item means with 99% confidence intervals. In Figure 1, dots and diamonds in the left panel are either purple for users or turquoise for non-users of online access. In the left panel of Figure 2 greener diamonds indicate higher means. In both figures, diamonds in the right panel show association strengths (correlation coefficients with 95% confidence intervals) between individual items and outcome. Fill color of those diamonds indicates the association strength and their direction; red diamonds mean strong and negative association, green diamonds mean strong and positive association and grey means weak association. Given that all associations were weak in absolute terms, the fill color of all diamonds appears subdued. Nevertheless, their relative strengths compared to each other can still be discerned by their position Beliefs about secured data privacy, sufficiency of received explanation or instruction and emotional benefits of online access showed both lower sample means and higher correlations than other beliefs with both use of online access and decisional conflict. In other words, they were strongly associated with both outcomes, but there still was room for improvement regarding these beliefs. They could therefore be regarded as highly relevant for use and decisional conflict regarding the use of online access. Specifically, those were participants’ beliefs that (i) viewing health information online is safe, (ii) when accessing health data online their privacy is guaranteed, (iii) they received sufficient explanation or instruction on how to use online access in and (iv) online access makes them feel less anxious.

Several beliefs about the impact of using online access on the role of the patient and the GP–patient relationship showed both lower sample means and higher correlations with and thereby relevance for decisional conflict, but not use: participants’ beliefs that by using online access, (i) they feel more involved in their healthcare, (ii) conversations with their GP feel more equal, (iii) they can prepare GP consultations better, (iv) their communication with the GP becomes better, (v) they have more information for making decisions about their health, (vi) they are more able to make decisions about their health that suit them and what they think is important and (vii) they are more able to make decisions about their health.

Other beliefs about the effects of online access were less relevant for use and decisional conflict as they were weakly associated with both. Those were beliefs about online access impacting the number of consultations, phone calls or personal encounters with the GP and practice staff, the amount of time invested by the patient and the patient’s feeling of overwhelm. Other beliefs were less relevant as they were associated with decisional conflict (but not use), yet the univariate distributions showed that participants were already convinced about these aspects, i.e. the beliefs showed no room for improvement. Those were beliefs about online access improving participants’ overview about their healthcare and appointments and increasing their ability to ensure the correction of mistakes in their medical files. Furthermore, also beliefs about the GP’s approval of patient online access and beliefs about perceived own ability to use online access were strongly correlated with decisional conflict (but not use) but were less relevant as participants were already convinced about these aspects. Preferring to hear information about personal health from the GP in person was also less relevant as it was neither correlated with decisional conflict nor use.

Explained variance was low with Nagelkerke *R*^2^ = 0.17 for the use of online access and adjusted *R*^2^ = 0.19 for decisional conflict. In response to finding low explained variance, we conducted post-hoc regression analyses to control for other factors. In addition to beliefs, all socio-demographic characteristics described under measurements in the methods section  were included in the regression. The analysis resulted in an increase of explained variance to Nagelkerke *R*^2^ = 0.22 for the use of online access and adjusted *R*^2^ = 0.23 for decisional conflict. As the increase was so small, we refrained from further exploring these socio-demographic factors.

## DISCUSSION

To our knowledge, this is the first study that provides detailed insight into the relevance of determinants for online access use and informed decision-making about whether to use online access. Beliefs about secured data privacy and sufficiency of explanation and instruction were highly relevant for the initial use of online access. Although concerns about complex and complicated access and privacy have been mentioned in reviews before (McGinn *et al*., 2011; Ose *et al*., 2017), it was frequently observed that many patients seemed willing to accept the risk of a security breach (Ose *et al*., 2017; Powell, 2017). Our study, however, suggests that those beliefs impact patients’ considerations to use online access. Therefore, privacy assurance, addressing potential patient misconceptions and fostering confidence in the safety of their data should be prioritized in measures to support patients’ adoption of and informed decision-making about online access.

Besides the belief that by using online access one feels less anxious, beliefs about the effects of online access were less relevant for use. This observation might seem contrary to earlier findings that describe the anticipation of benefits as facilitators for use (Powell, 2017; Crameri *et al*., 2020). However, those reviews included studies focusing on determinants of both initial and long-term ongoing use and engagement with a patient portal, while in our study use was defined as using online access at least once. Likely, determinants for initial and sustained long-term use differ. Assured privacy and sufficient instruction might be the most relevant prerequisites for the initial use of online access, while more or other determinants become relevant for sustained use. Additionally, evaluation of some effects might vary across participants based on individual preferences. For example, the expectation that online access leads to fewer consultations with the GP might be regarded as beneficial because time-saving for some, and undesirable for others who prefer personal encounters with the GP. In other words, whether a belief about an effect works as a facilitator or barrier for the use of online access might differ between patients, which could explain the low relevance of beliefs about effects in the overall sample.

In contrast to use itself, however, beliefs about effects seem to be highly relevant for patients to make an informed decision regarding use. Next to the beliefs that showed relevance for use, beliefs about effects, i.e. beliefs that the use of online access would improve the communication with the GP and the patient’s ability to participate in healthcare, were most relevant in explaining decisional conflict regarding use. Sufficient relevant knowledge about the benefits and risks of all alternative options and being clear about personal values are described as key requirements for enabling informed decision-making (Bekker *et al*., 1999). Building on this definition, our findings suggest that information about the possible effects of online access is necessary to endow patients with relevant knowledge for an informed decision regarding use. Moreover, following the assumption that the evaluation of effects might vary between patients, our results stress the importance of patients recognizing their personal preferences. Therefore, communicating potential effects and supporting patients’ exploration of individual preferences, e.g. during a consultation with the GP, is recommended to facilitate patients’ informed decision-making about whether to use online access.

Explained variance for use of online access (Nagelkerke *R*^2^ = 0.17) and decisional conflict (adjusted *R*^2^ = 0.19) was rather low, which might have several reasons that are not mutually exclusive: first, the number of the categories used to measure the determinants does not equal the number of categories used to measure the outcomes, which makes it impossible to explain 100% of the variance (Sutton, 1998). However, it can be assumed that the attributable constraint on predictive power is small.

Second, the low explained variance might indicate the influence of some factors not included in the analyses. However, the items in this study were based on findings from previous research and a preceding interview study among the same target group (Thielmann *et al*., 2023). Considering these thorough preparatory steps, we think it is unlikely that we missed psychological determinants that would explain large parts of variance. Even with the inclusion of socio-demographic characteristics in post-hoc regression analyses, there was only a marginal increase in explained variance. Consequently, it is improbable that influential factors were omitted in the analysis. Instead, findings from the post-hoc analysis suggest the absence of distinct sub-groups of patients—categorized by factors like age or education—that are more or less likely to use online access. This observation indicates that predicting a patient’s preference to use online access may not be essential. Instead, it seems important to recognize the variability in individual preferences and decisions and to present every patient with the opportunity to use online access.

Third, building on this reasoning, the low explained variance might imply that (the decision regarding) the use of online access is less the result of cognitive thought processes or patient characteristics but instead depends on if and how patients receive the opportunity to use online access. Typically, individuals tend to adhere to the status quo rather than doing something different as it might require cognitive or practical effort (Voyer, 2015). Effectively promoting the use of online access and facilitating informed decision-making might hinge on strategies that raise awareness, enhance convenience and reduce the effort required for use. One approach could be general practices of sending letters to all patients, providing information about online access, steps to acquire it and contact details for any additional questions patients may have. Similarly, the use of a more intensive promotional strategy through various means (e.g. posters in waiting areas, automated messages on the practice’s telephone system, letter mailings, staff speaking to the patient in the office or over the telephone) was found to increase the odds of portal adoption almost threefold (Yamin *et al*., 2011). As broader implementation strategies that focus on both patients and healthcare providers were found to result in higher user rates (Lyles *et al*., 2020), we recommend future research to explore the role of and barriers for GP and practice staff in facilitating and promoting online access for patients.

### Strengths and limitations

The strength of this study is the thorough preceding literature and qualitative research aimed at identifying all possibly relevant determinants. A few limitations of this study have to be mentioned. First, the cross-sectional design of the study does not allow for causal interpretations of the results. Second, the digital and health literacy scores of the study population were rather high, possibly reflecting a selection bias of this study conducted exclusively with members of an online survey panel. However, the characteristics are in line with findings within the Dutch population, as 63.6% of adults were classified as having sufficient health literacy in 2018 (Heijmans *et al*., 2018) and almost 80% had basic or above average overall digital skills in 2019 (Eurostat, 2021). Third, the CIBER approach does not analyze interactions between determinants and examine possible confounders. However, in line with the aim of the study, we selected CIBER as the combination of both univariate distributions of determinants and their associated strengths with the outcomes of interest to provide interesting indications for strategies supporting patients in the use of online access.

### Practical implications

Future efforts to overcome challenges in the adoption of patient online access should emphasize secured data privacy and the provision of sufficient instruction and explanation. Communicating potential effects and facilitating the exploration of individual patient preferences is recommended to support patients’ informed decision-making regarding the use of online access. As the relevance of psychological determinants seems limited, we advise primarily ensuring all patients are given the opportunity to get online access and to minimize the required effort.
